# Chest Radiography Scores for Predicting the Severity of Respiratory Status in Newborns Weighing More Than 1,500 g

**DOI:** 10.7759/cureus.77315

**Published:** 2025-01-12

**Authors:** Kisho Asuka, Masashi Zuiki, Tomohiro Hasegawa, Rei Takada, Madoka Konishi, Akio Yamano, Eisuke Ichise, Kanae Hashigushi, Tatsuji Hasegawa, Tomoko Iehara

**Affiliations:** 1 Pediatrics and Neonatology, Kyoto Prefectural University of Medicine, Kyoto, JPN

**Keywords:** acute respiratory failure (arf), brixia score, neonatal intensive care unit, rale score, x-ray radiography

## Abstract

Background

Acute respiratory failure (ARF) may occur in neonates. Chest radiography is commonly used to evaluate the severity of ARF; however, the application of quantitative scales in clinical practice in neonatal intensive care units is uncommon. This study aimed to assess the usefulness of two semi-quantitative radiographical scales, the Brixia and radiographic assessment of lung edema (RALE) scores, in newborns weighing more than 1,500 g.

Methods

Newborns weighing > 1,500 g who received invasive respiratory support with arterial lines between January 2010 and October 2023 were enrolled in this study (n = 98; gestational age, 35.6 ± 3.1 weeks; birthweight, 2,321 ± 600 g). We investigated the correlation between the Brixia or RALE scores and the oxygen index (OI), alveolar-arterial oxygen gradient (A-aDO_2_), and ventilation index (VI). Furthermore, the cut-off points of the two radiographic scores for the prediction of these respiratory indices were determined.

Results

All respiratory indices correlated with the Brixia (OI: *r *= 0.71, *p < *0.001; A-aDO_2_:* r *= 0.74, *p < *0.001; VI:* r *= 0.56, *p < *0.001) and RALE scores (OI: *r *= 0.78, *p < *0.001; A-aDO_2_:* r *= 0.82, *p < *0.001; VI:* r *= 0.60, *p < *0.001). Additionally, the receiver operating characteristic curve showed that the radiographical scores had a statistically significant ability to predict respiratory index values.

Conclusion

Brixia and RALE scores are useful predictive markers of acute respiratory failure in infants weighing >1,500 g. These chest radiography scores may be good predictors of respiratory status and have wider clinical applications in neonatal care.

## Introduction

Acute respiratory failure (ARF) is the most common complication observed in newborns admitted to neonatal intensive care units (NICUs). Most neonatal pulmonary diseases are diagnosed and managed based on chest radiography (CXR); however, the lack of standardization and objectivity makes it challenging to accurately assess the severity of ARF using CXR. The oxygenation index (OI), alveolar-arterial oxygen gradient (A-aDO_2_), and ventilation index (VI) are often used clinically to identify ventilated infants with ARF, evaluate the response to treatment and severity of ARF, and predict mortality or successful extubation [1-5]. However, the disadvantages of employing these respiratory indicators in the NICU include the necessity of invasive arterial puncture to acquire a blood gas sample.

The Brixia and radiographic assessment of lung edema (RALE) scores have recently been identified as useful tools based on CXRs for assessing the severity of respiratory distress in patients [6,7]. We have previously reported an association between these indices and hypoxic respiratory failure in very low birth weight infants (VLBWIs) [8]. However, whether these scores are also useful in more mature newborns remains unclear, because the causes of respiratory failure vary with lung immaturity.

We hypothesized that CXR findings could serve as predictors of ARF severity in term and late preterm infants. The objective of this study was to evaluate the relationships between Brixia or RALE scores and OI, A-aDO_2_, and VI in neonates weighing ≥ 1,500 g, as well as to identify the optimal cut-off values for these two radiographical scores in predicting oxygenation and ventilation status.

## Materials and methods

**Figure 1:**
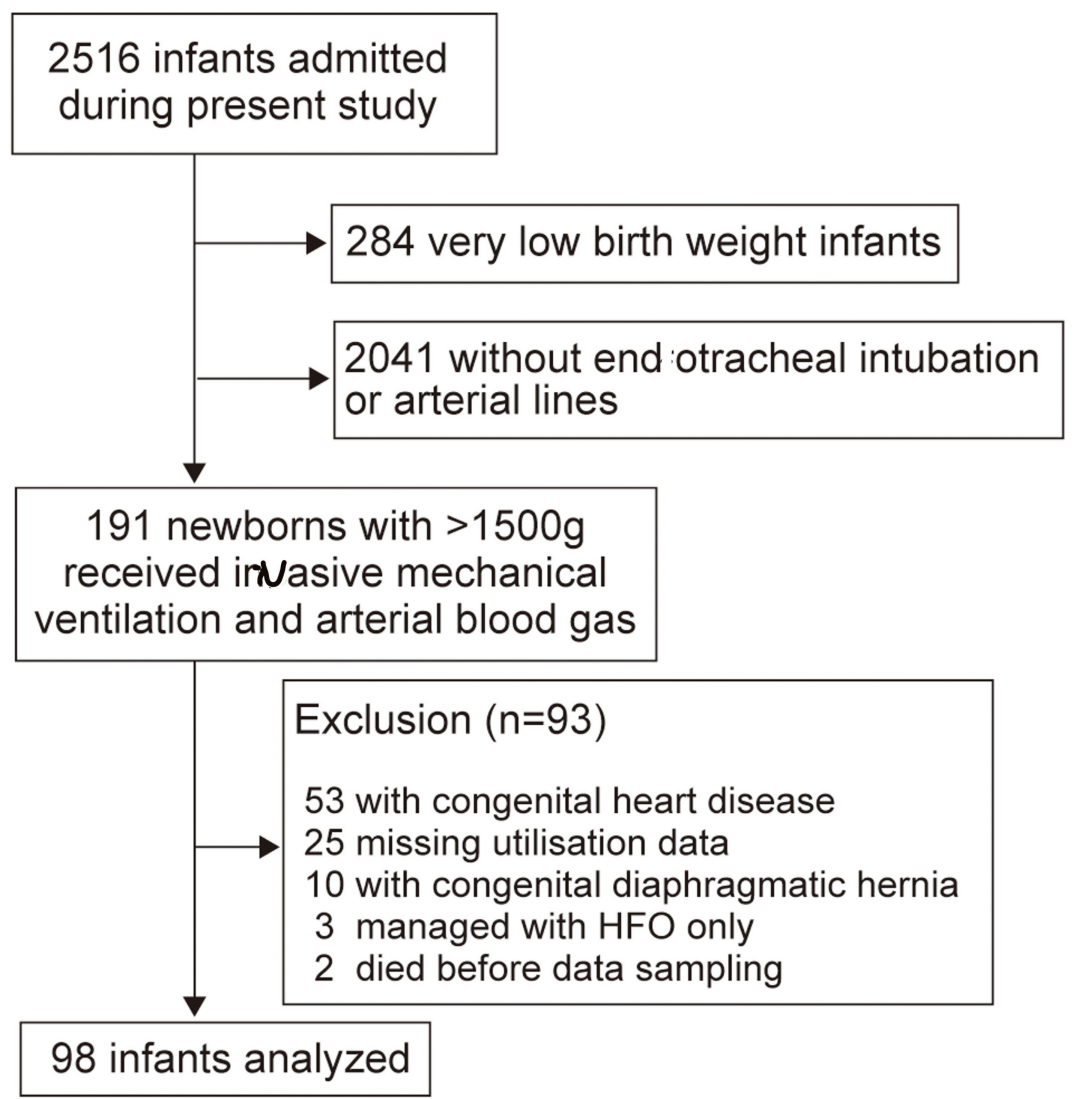
Flow diagram showing the number of included infants HFO: High-frequency oscillation

This single-center cross-sectional study was approved by the Clinical Ethics Committee of the University Hospital, Kyoto Prefectural University of Medicine, Kyoto, Japan (ERB-C-2794). Ethical approval was obtained to implement an opt-out consent process for all eligible infants born in the participating unit. Parents of eligible infants were provided with the option to decline the inclusion of their infants’ data in this study. This study included infants weighing ≥ 1,500 g who received mechanical ventilation using synchronous intermittent mandatory ventilation with arterial lines. These infants had been admitted to the NICU by the age of 14 days, between January 2010 and August 2024 (Figure 1). The attending neonatologist performed further clinical interventions including endotracheal intubation, arterial catheter insertion, and/or mechanical ventilation initiation. Patients with only high-frequency oscillation data, congenital heart disease that affected the systemic circulation, or congenital diaphragmatic hernia were excluded from the study. The study used data from the electronic medical records that encompassed pre- and perinatal clinical details, CXR findings, respiratory settings, and arterial blood gas analysis results.

The methodology for selecting the CXR images used in the study and details of the Brixia and RALE scores have been previously described [6-8]. The initial CXRs performed simultaneously with arterial blood sampling during mechanical ventilation were used in the study (one per patient). This study did not use radiographs to examine conditions such as symptomatic patent ductus arteriosus, pneumothorax, and persistent pulmonary hypertension of the newborn, or immediately following surfactant administration.

The Brixia scoring system categorizes CXRs into six distinct sections, each assigned a score that reflects the presence of interstitial and alveolar infiltrates, with scores ranging from 0-18. Each radiograph was divided into quadrants to determine the RALE score. The products of the consolidation and density scores assigned to each quadrant were summed to yield the final RALE score (ranging from 0-48). The methods of calculating the respiratory indices were as follows:

OI = fraction of inspired oxygen (FIO_2_) × mean airway pressure (MAP) × 100/partial pressure of oxygen (PaO_2_); A-aDO_2_= FiO_2_ × 713 - partial pressure of carbon dioxide (PaCO_2_)/0.8 −PaO_2_; VI = peak inspiratory pressure (PIP) × PaCO_2_ × ventilation frequency/1000

First, the CXR scores were evaluated by two neonatologist reviewers who were blinded to the clinical data. Next, the correlation between the two radiographical methods and the OI, A-aDO_2_, and VI were assessed. Finally, a receiver operating characteristic (ROC) curve analysis was performed to determine the optimal cut-off points of the Brixia and RALE scores for predicting these indices (OI: ≥2.5, ≥5, and ≥7.5; A-aDO_2_: ≥100, ≥200, and ≥300; VI ≥10, ≥20, and ≥30).

Statistical analyses

Each correlation was analyzed using Spearman's correlation coefficient. The Bland-Altman technique was used to assess the measurement agreement by plotting the difference (D) using the average for each measurement. The 95% limits of agreement (D ± 2 standard deviation (SD)) depicted a range that included most of the differences in the methods. ROC curve analysis was used to assess the predictive value of different severity indices for the outcome in patients with ARF and to determine the best cut-off values for the radiographical scores. P-values < 0.05 were considered statistically significant. All statistical analyses were performed using EZR software (Saitama Medical Center, Jichi Medical University, Saitama, Japan).

## Results

In total, 98 newborns met the inclusion criteria. The enrolled infants had a mean gestational age of 35.6 ± 3.1 weeks and a mean birth weight of 2,321 ± 600 g. The mean Apgar scores at 1 and 5 min were 4.8 ± 2.6 and 6.5 ± 2.2 points, respectively. The median number of days at measurement was 1 (interquartile range, 1-3). The reasons for intubation were transient tachypnea of the newborn in 28 cases (29%), respiratory distress syndrome in 22 cases (22%), meconium aspiration syndrome in seven cases (7%), and dry lung syndrome or pulmonary hypoplasia in four cases (4%) (Table 1).

**Table 1:** Descriptive characteristics of the enrolled patients Values are represented as means (±standard deviations) or median (interquartile range) unless specified otherwise.

Parameter	Clinical data (n=98)
Gestational age, weeks	35.6 ± 3.1
Birth weight, g	2,321 ± 600
Male / Female, n (%)	47 (48) / 51 (52)
Cesarean section, n (%)	52 (53)
Twin birth, n (%)	10 (10)
Apgar score at 1 min	4.8 ± 2.6
Apgar score at 5 min	6.5 ± 2.2
Days of measurements, days	1 (1-3)
Reason for intubation	
Transient tachypnea of newborns, n (%)	28 (29)
Respiratory distress syndrome, n (%)	22 (22)
Meconium aspiration syndrome, n (%)	7 (7)
Dry lung syndrome or Pulmonary hypoplasia, n (%)	4 (4)
Operation, n (%)	21 (21)
Asphyxia or encephalopathy, n (%)	13 (13)
Others, n (%)	3 (3)

**Figure 2:**
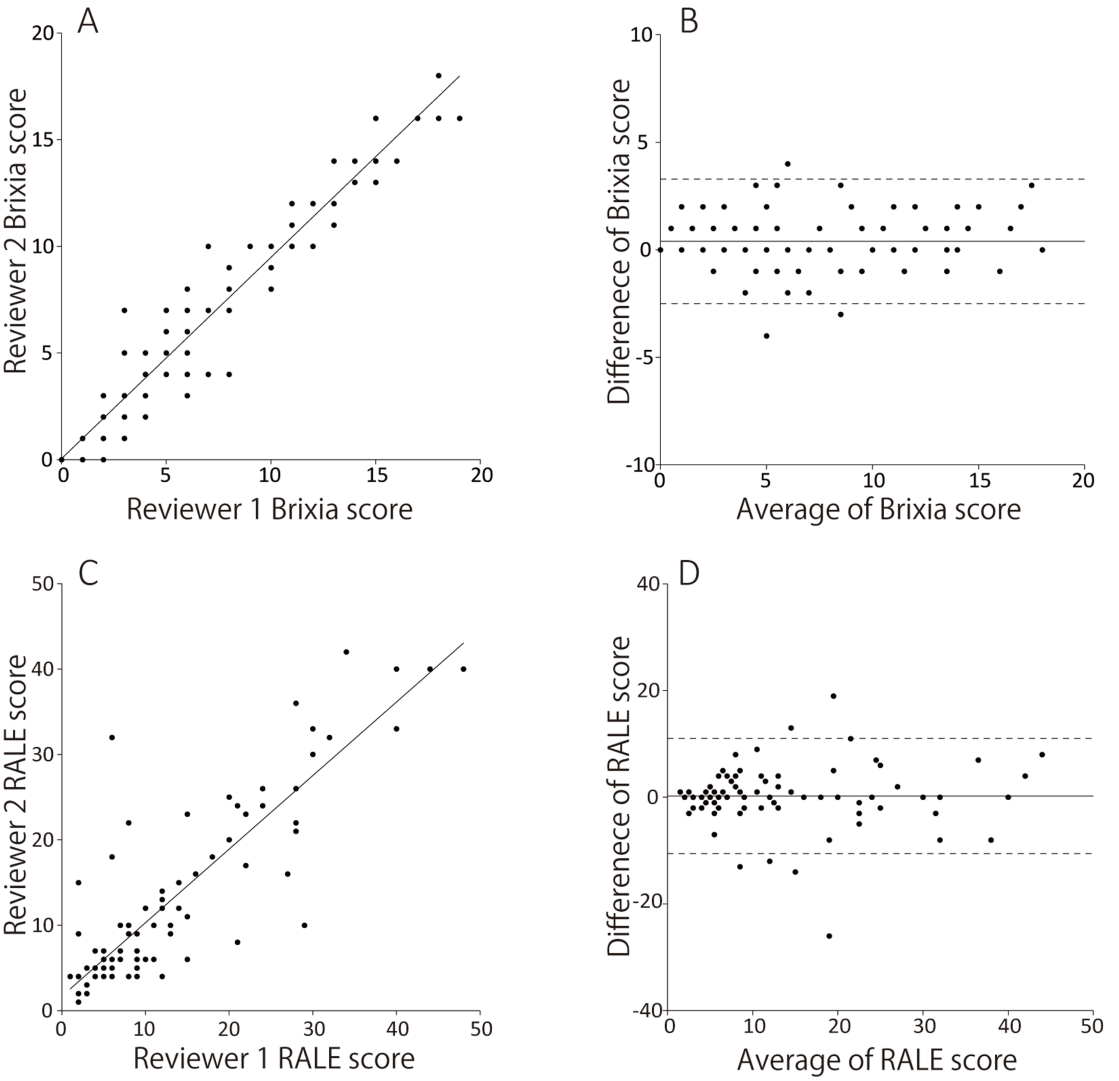
Scatter plots (A and C) and Bland–Altman plots (B and D) show agreement between the two independent reviewers for the Brixia (A and B) and radiographic assessment of lung edema (RALE) scores (C and D). The Brixia and RALE scores of the two reviewers correlated positively (Brixia: r = 0.95, p < 0.001; RALE: r = 0.88, p < 0.001). In the Bland–Altman analysis, the horizontal lines denote the estimated bias (solid line) and 95% limits of agreement (dotted line). The 95% limits of agreement were as follows: Brixia score: 0.4 ± 1.4 and RALE score: 0.2 ± 5.4.

Figure 2A and Figure 2C show that the Brixia and RALE scores of the two reviewers correlated positively (Brixia: r = 0.95, *p* < 0.001; RALE: r = 0.88, *p* < 0.001). The results of the Bland-Altman analysis are shown in Figure 2B and Figure 2D. Good agreement was observed between the measurements, with a mean bias of 0.4 (Brixia) and 0.2 (RALE); the 95% limits of agreement (±2 SD) were 1.4 (Brixia) and 5.4 (RALE).

**Figure 3:**
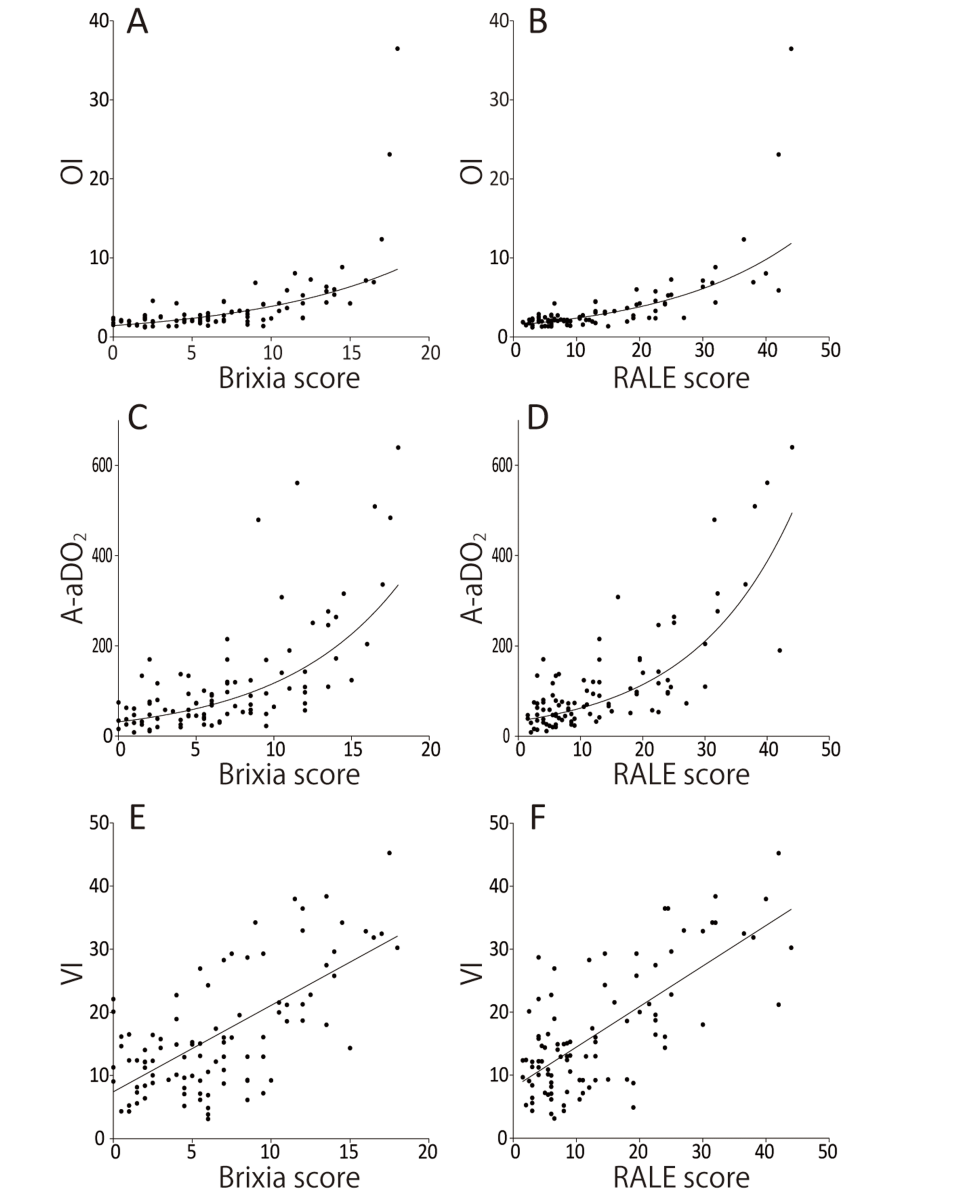
Regression analysis for the relationship between the two radiographic scores and oxygen index (OI), alveolar-arterial oxygen gradient (A-aDO2), or ventilation index (VI). These respiratory indices correlated positively with the Brixia (OI: r = 0.71, p < 0.001; A-aDO_2_: r = 0.74, p < 0.001; VI: r = 0.56, p < 0.001) and RALE scores (OI: r = 0.78, p < 0.001; A-aDO_2_: r = 0.82, p < 0.001; VI: r = 0.60, p < 0.001).

Figure 3 shows that a significant positive correlation was observed between each respiratory index and the Brixia (OI: r = 0.71, *p* < 0.001; A-aDO_2_: r = 0.74, *p* < 0.001; VI: r = 0.56, *p* < 0.001) and RALE scores (OI: r = 0.78, *p* < 0.001; A-aDO_2_: r = 0.82, *p* < 0.001; VI: r = 0.60, *p* < 0.001). Furthermore, ROC curve analysis showed that graphical scores had a statistically significant ability to predict the OI, A-aDO_2_ and VI. The optimal cut-off points of two radiographical scores for predicting these respiratory indices are shown in Table 2.

**Table 2:** The receiver operating characteristic curve analysis for predicting respiratory indices AUC, area under the curve; A-aDO_2_, alveolar-arterial oxygen gradient; CXR, chest radiography; CI, confidence interval; OI, oxygenation index; RALE, radiographic assessment of lung edema; VI, ventilation index

CXRs score	Respiratory index	AUC	95% CI	Cut-off value	Sensitivity	Specificity
Brixia	2.5 ≤ OI	0.85	0.77 - 0.92	7	80%	78%
	5 ≤ OI	0.97	0.94- 1.0	11	92%	93%
	7.5 ≤ OI	0.96	0.90 - 1.0	12	85%	100%
	100 ≤ A-aDO_2_	0.84	0.77 - 0.92	7	76%	82%
	200 ≤ A-aDO_2_	0.94	0.88 - 0.99	9	81%	93%
	300 ≤ A-aDO_2_	0.93	0.85 - 1.0	11	82%	88%
	10 ≤ VI	0.68	0.66 - 0.92	6	79%	57%
	20 ≤ VI	0.84	0.74 - 0.95	9	88%	72%
	30 ≤ VI	0.95	0.91 - 0.96	12	91%	92%
RALE	2.5 ≤ OI	0.86	0.78 - 0.94	13	88%	78%
	5 ≤ OI	0.98	0.95 - 1.0	20	87%	100%
	7.5 ≤ OI	0.99	0.97 - 1.0	32	97%	100%
	100 ≤ A-aDO_2_	0.84	0.75 - 0.92	10	71%	85%
	200 ≤ A-aDO_2_	0.95	0.893 - 0.99	25	89%	100%
	300 ≤ A-aDO_2_	0.96	0.893 - 1.0	32	98%	88%
	10 ≤ VI	0.69	0.58 - 0.79	11	79%	54%
	20 ≤ VI	0.83	0.72 - 0.94	15	84%	79%
	30 ≤ VI	0.98	0.96 - 1.0	24	93%	100%

## Discussion

In this study, we demonstrated that Brixia and RALE scores correlated with each respiratory index in term and late preterm infants who underwent mechanical ventilation. Moreover, we determined the cut-off values of the two radiographic scores for predicting oxygenation and ventilation status.

In the past, the Brixia and RALE scores were documented as quantifiable and objective indicators that may assist in addressing various clinical challenges, such as the estimation of acute lung injury or pneumonia, or assessment of the need for mechanical ventilation [9-17]. Although reports on these radiographical scores remain rare in the neonatal field, we recently demonstrated their potential as predictive markers of oxygenation status in intubated VLBWIs [8]. Relatively mature newborns develop respiratory disease for various reasons, including transient tachypnea of the newborn or meconium aspiration syndrome, which differ from those in VLBWIs. In addition, the lungs of infants admitted to NICUs are still developing. Preterm infants born between 22 and 32 weeks of gestation are reaching the end of the canalicular and saccular stages of lung development. Subsequently, alveolar proliferation and development occur during the alveolar phase after 36 weeks [18]. Therefore, CXR scores are necessary in infants weighing more than 1,500 g, not just in VLBWIs. We recommend the use of the cut-off values outlined in this study as a reference for the respiratory management of term or late preterm infants.

The gold standard for evaluating respiratory status is computed tomography imaging. However, it is often problematic to perform in ventilated neonates in NICUs because of the need for transfer to a dedicated device, and radiation exposure. Although several studies have investigated the use of lung ultrasound for respiratory management of newborns, the presence of air in the lungs is a limitation in ultrasound scanning due to various artifacts [19,20]. Electrical impedance tomography is a new clinical imaging tool for monitoring the distribution of ventilation, but its use in newborns is limited [21]. CXR is the most valuable and commonly used imaging technique for the assessment of neonatal ARF. Therefore, we believe that CXR scores can contribute to the assessment of respiratory status in newborns, and have the potential for broader clinical use in NICUs.

This study had several clinical implications. First, despite advances in neonatal intensive care, the clinical application of respiratory monitoring techniques including pulse oximetry and capnography continues to be restricted to neonates [22,23]. We suggest that evaluations combining these methods with radiographical scores could help reduce the frequency of blood sampling and invasive procedures and improve the accuracy of diagnosis of neonatal respiratory failure, as well as the workload and cost of NICUs. Furthermore, neonatal respiratory status can be objectively and quantitatively assessed using these radiographical scoring systems. Therefore, these scores can contribute to the evaluation of therapeutic effects or observation of changes in respiratory status.

Our study had a few limitations. Alterations in pulmonary blood flow and adjustments to ventilator settings can influence CXRs. Furthermore, our findings may have been constrained by the potential bias inherent in the single-center study design. Finally, retrospectively collected data may not be accurately matched with data from the same temporal reference point. Future studies with stricter protocols are required to clarify the effectiveness of this radiographical scoring system for neonates.

## Conclusions

In this study, we investigated the correlation between Brixia or RALE scores and the respiratory indices in newborns weighing more than 1,500 g. In addition, the cut-off values of these chest radiography scores for the prediction of oxygenation and ventilation status in ventilated infants were determined. We believe that these radiographic parameters can contribute to assess the respiratory status of newborns and have the potential for broader clinical use in NICUs.
